# Penetrating penile injury due to the splintering of the floorboards in the gymnasium

**DOI:** 10.1002/iju5.12290

**Published:** 2021-05-04

**Authors:** Tomoyuki Kaneko, Akihiko Sakamoto, Takahiro Yoshida, Kazuki Yanagida, Itsuki Yoshimura, Kanade Hagiwara, Masaki Kimura, Yukio Yamada, Tohru Nakagawa

**Affiliations:** ^1^ Department of Urology Teikyo University School of Medicine Tokyo Japan

**Keywords:** fitness centers, genitalia, penetrating, penis, wounds and injuries

## Abstract

**Introduction:**

We present the case of a patient with penetrating penile injury caused by splintering floorboards in a gymnasium.

**Case presentation:**

A 24‐year‐old man was brought to the emergency department of our hospital because of an unintentional penetrating penile injury sustained while playing volleyball at a gymnasium. He dove into the wooden floor to fly‐receive the ball. When sliding with his abdomen on the floor, a wooden splinter from the floorboard stuck from the base of his penis to near the glans penis. The splinter was gently removed without bleeding under local anesthesia.

**Conclusions:**

Splintering floorboards in gymnasiums can cause serious trauma, including penile injuries. Health‐care workers and users of public facilities, such as gymnasiums, should be aware of the accident risk associated with wooden floors.

**Keynote message**

Splintering floorboards in gymnasiums can cause serious trauma, including penile injuries. Health‐care workers and users of public facilities, such as gymnasiums, should be aware of the accident risks associated with wooden floors.

## Introduction

Genital trauma is commonly caused by blunt injuries. Penetrating injury to the external genitalia is relatively rare, and most commonly caused by incidents related to firearms.^1^ Penetrating injury to the penis is also uncommon, and the causes are gunshot wounds, self‐ or non‐self‐mutilation, animal or human bites, and foreign‐body insertion for autoerotic reasons.^2^, ^3^, ^4^, ^5^, ^6^, ^7^, ^8^ We present the case of a patient with penetrating penile injury caused by splintering floorboards in a gymnasium.

## Case report

**Fig. 1:**
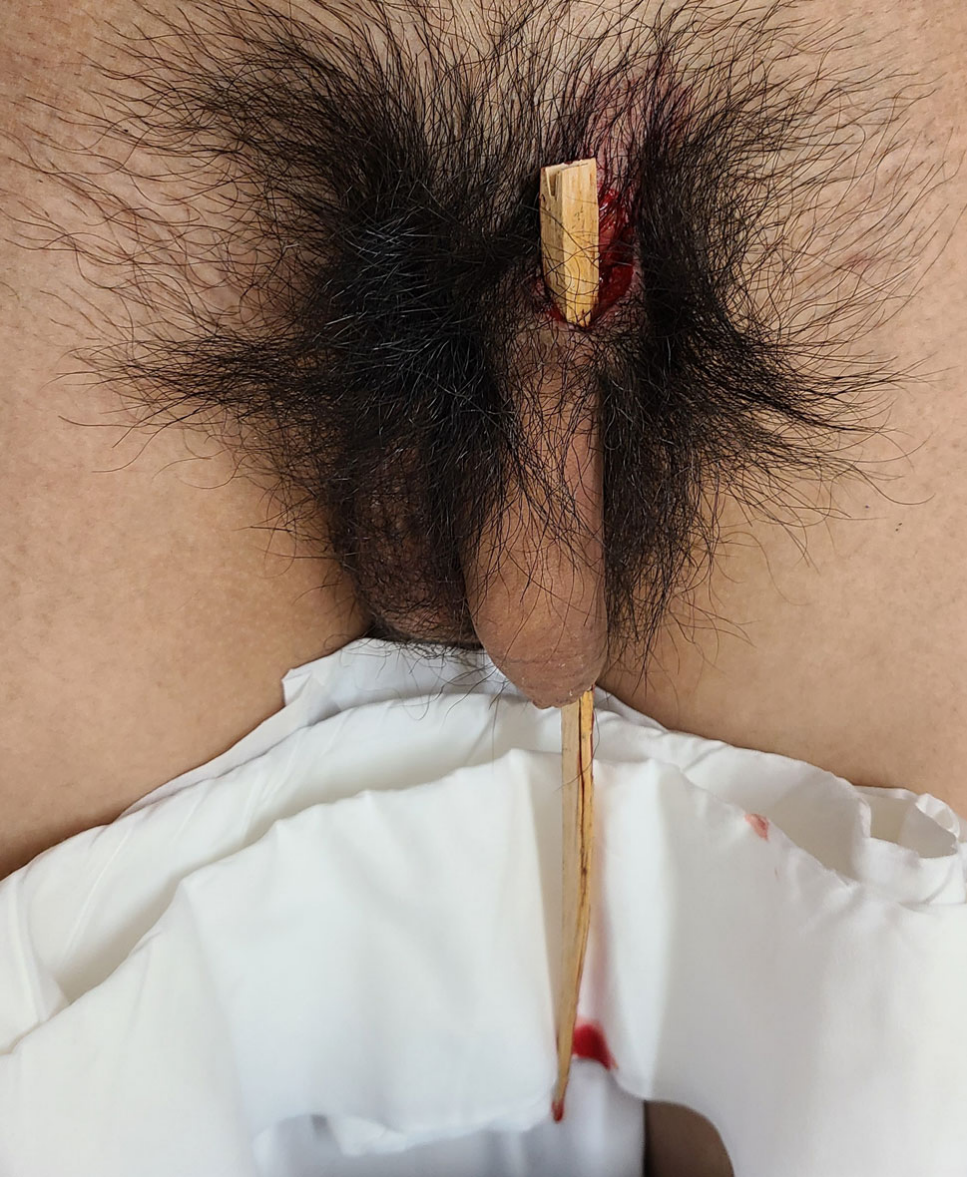
Photograph showing penetrating penile injury with a wooden splinter of the floorboards.

**Fig. 2:**
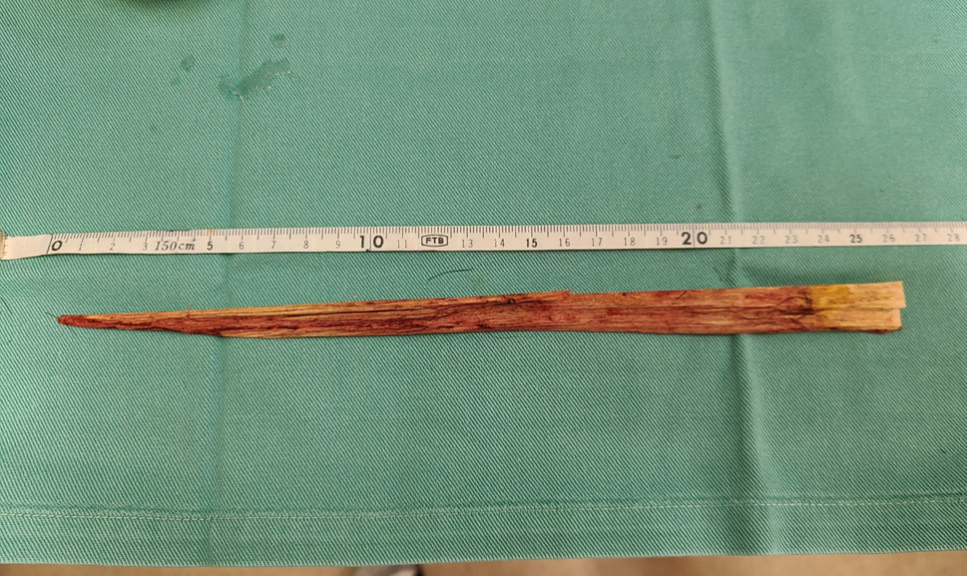
Photograph of a wooden splinter of the floorboards removed from the patient's body.

A 24‐year‐old man was brought to the emergency department of our hospital via ambulance because of an unintentional penetrating penile injury sustained while playing volleyball at a gymnasium. During a practice session for the volleyball club, the patient dove into the wooden floor with the top half of his body to fly‐receive the ball. When sliding with his abdomen on the floor, he experienced pain around his genitalia. Thereafter, he noticed that a wooden splinter from the floorboard was stuck in his pubic area. On physical examination, the patient was alert, and his vital signs were normal. The wooden splinter was sticking from the base of his penis, penetrating the subcutaneous tissue of the foreskin and came out from near the glans penis (Fig. 1). Based on the macroscopic findings, it was judged that the damage was in the superficial subcutaneous layer. Under local anesthesia, the splinter was gently removed without bleeding. The wooden splinter was about 26 cm in length and 2 cm in width (Fig. 2). After saline irrigation, a penrose drain was inserted in the wound. The skin was closed with 3‐0 nonabsorbable interrupted sutures. The patient was sent home after the administration of tetanus toxoid with oral antibiotics. The drain was removed on the following day, and his recovery period was uneventful.

## Discussion

To our knowledge, this is the first reported case of penetrating penile injury caused by splintering of the floorboards in the gymnasium. On 29 May 2017, the Consumer Safety Investigation Commission of Japan published the investigation report about accidents wherein the subject sustained serious injury because of conditions, such as the sticking of splintering floorboards of the gymnasiums in their abdomens (Investigation Report into the Causes of Accidents as Stipulated in Article 23 of the Consumer Safety Act, https://www.caa.go.jp/policies/council/csic/report/report_010/pdf/report_010_171228_0001.pdf, accessed on 1 February 2021). They collected data of the subjects who had sustained accidental injuries that were recorded in the accident information data bank of the Consumer Affairs Agency and press information; a total of seven accidents of the same or similar type were reported between 2006 and 2015. Of these, six occurred during volleyball and one occurred during futsal. Three subjects sustained abdominal injury, three sustained thoracic injury, and one had a left thigh injury. In one case of abdominal injury sustained during volleyball, the patient was practicing a “flying receive” without using a ball, and when he/she slid on the floor, part of the floorboards stuck into his/her abdomen. A splinter entered the lower left nipple and punctured the stomach, transverse colon, jejunum, and mesentery. A laparotomy was performed in order to separate and excise the 34‐cm long splinter. In another case of thoracic injury that occurred during a futsal game, the patient, who was the goalkeeper, dove to intercept the ball; when sliding with his/her back on the floor, part of the floorboard stuck in his/her back. A wooden fragment was sticking from the top of their shoulders, and it had punctured his/her lung and reached his/her liver. However, despite serious accidents, such events are not widely known even among health‐care professionals. In Japan, most gymnasium floors are made of wood. Wooden materials, in response to changes in ambient temperature and humidity, may absorb moisture present in the air and swell; they then release this moisture and shrink. If wood is dried quickly, deviations may occur in the distribution of the moisture inside the wood, causing strain between the significantly shrunk area and other areas, potentially causing cracks. The cause of the defects occurring in the floorboards is not only the deterioration arising from the use of wooden floors, but also the excessive moisture absorption or drying at the various stages of use. In addition to deterioration that accompanies the use of wooden floors and moisture, other factors that may cause defects in floorboards are foreign matter, such as earth and sand, hard sharp objects, and heavy objects. The wooden floorboards of the gymnasium should be kept clean with daily cleaning. In addition, it is important to minimize the impact of moisture. One should remember that wet mopping and waxing needs to be avoided to prevent floorboard defects. The deterioration of the wooden floors of gymnasiums is inevitable owing to the constant use for exercise and other events; therefore, regular maintenance and management is necessary. In addition to preventing defects from occurring in the floorboards, it is necessary to appropriately deal with defects in case they appear. It is noteworthy that accidents also occur in new gymnasiums; therefore, the risk of the same kind of accident is considered to be present in all wooden‐floored gymnasiums, irrespective of the number of years of use. It is necessary for the owner and manager of the gymnasium to perform their duties with an awareness regarding the risk of injuries and for users of the gymnasium to be aware of the accident risk. In countries other than Japan, only one similar case has been reported in Brazil and one in the USA (National Training Center shared court Accident Cause Investigation Committee Report, https://www.jpnsport.go.jp/hpsc/Portals/0/news/pdf/170830_houkoku.pdf, accessed on 1 February 2021). These incidences may be attributable to the materials used for making the gymnasium floor or the climate difference. Urologists should be aware that such genital trauma can occur in public facilities, such as gymnasiums. In penetrating penile trauma, nonoperative management is recommended for small superficial injuries with intact Buck's fascia.^2^ Surgical exploration as well as debridement of the necrotic tissue is recommended in more significant penetrating penile injuries. The principles of care are debridement of devitalized tissue, hemostasis, removal of foreign bodies, and the diversion of urine in selected cases. Although the surgical approach depends on the site and extent of the injury, a subcoronal incision with penile degloving usually gives good exposure. A defect in the tunica albuginea should be closed after copious irrigation. In case of excessive tissue loss, the defect can be repaired with a patch, either from an autologous saphenous vein or from a xenograft.^9^

## Conflict of interest

The authors declare no conflict of interest.
